# Implementation of AI in oncology: a systematic review of educational and clinical integration in global contexts

**DOI:** 10.3389/fdgth.2026.1764390

**Published:** 2026-02-04

**Authors:** Talia Tene, Diego Fabián Vique López, Paulina Elizabeth Valverde Aguirre, Kathy Violeta Serrano Avalos

**Affiliations:** 1 Department of Chemistry, Universidad Técnica Particular de Loja, Loja, Ecuador; 2 Facultad de Salud Pública, Escuela Superior Politécnica de Chimborazo (ESPOCH), Riobamba, Ecuador; 3 Facultad de Ciencias, Escuela Superior Politécnica de Chimborazo (ESPOCH), Riobamba, Ecuador

**Keywords:** artificial intelligence, cancer training, ChatGPT, clinical integration, education, oncology, systematic review

## Abstract

**Introduction:**

The global burden of cancer, coupled with inequalities in access to healthcare, has intensified interest in artificial intelligence (AI) as a transformative force in oncology. This study systematically analyzes how AI is being integrated into oncology education and clinical practice globally, highlighting its role in training the next generation of healthcare professionals.

**Methodology:**

A systematic review was conducted following the PRISMA and PICO guidelines, using data from PubMed, Scopus, and IEEE Xplore. Studies from 2019 to 2024 were selected based on empirical evaluations of AI-based educational interventions in oncology. Educational outcomes were extracted as performance (e.g., accuracy, test scores, comprehension/quality ratings), engagement/acceptability (e.g., usability or satisfaction ratings, completion), and skills development (e.g., task-based procedural metrics), as reported by each study. A total of 16 studies met the inclusion criteria after a rigorous selection and eligibility verification process.

**Results and discussion:**

The findings reveal that AI tools, especially chatbots based on large language models (e.g., ChatGPT), are increasingly being used in the education of both patients and professionals. Positive outcomes include improved knowledge retention, diagnostic reasoning, and engagement.

**Conclusion:**

AI is transforming cancer education through personalized and accessible learning experiences. Strategic integration, rigorous validation, and equitable access are essential for sustainable impact across diverse global settings.

## 1 Introduction

The growing cancer crisis has sparked global interest in innovative solutions to improve healthcare (1), especially in contexts where healthcare infrastructure faces critical limitations. Simultaneously, the last decade has witnessed the rapid emergence of artificial intelligence (AI) as a transformative tool in oncology (2). AI, which includes techniques such as machine learning, deep learning, and natural language processing (3), offers unprecedented capabilities for analyzing large volumes of complex medical data to detect (4), diagnose, predict, and select cancer treatments (1, 5). Researchers and clinicians agree that AI has the potential to revolutionize cancer care by improving clinical decision-making and enabling more personalized, data-driven interventions (6).

Historically, AI in medicine has come a long way (7), from experimental expert systems to the sophisticated algorithms that now fuel advances in diagnosis and treatment (1, 8). Early AI systems were logic-based, following “if-then” rules, but showed limited results outside of research settings (9). In contrast, contemporary technologies rely on technical algorithms that learn directly from real-world data, capable of identifying complex patterns that may even escape the trained eye. This progress has coincided with substantial improvements in computing power and the availability of big data in healthcare (4, 10). In precision oncology, for example, AI can now integrate multi-omics data (genomic, proteomic, and metabolomic) with clinical variables to guide therapeutic decisions (11), offering unprecedented sophistication compared to previous technologies (4).

The application of AI spans the entire spectrum of cancer care (12). In the field of diagnostics, AI-based image analysis systems have proven to match or even surpass human specialists in specific tasks (13), which can accelerate detection and reduce bottlenecks in pathology and radiology departments. Beyond medical imaging (14), AI systems have also been developed that can improve cancer detection through the analysis of clinical texts (15), genetic sequences (4), or laboratory data (16).

In the field of prognosis and treatment planning (5), the exponential growth of high-dimensional data—from genomic sequencing to digitized radiological images—has enabled the design of machine learning models to predict disease progression and select the most appropriate treatment. In radiation oncology, for example, AI tools are used to analyze medical (radiomic) images and predict tumor radiosensitivity (17), which could allow for personalized radiation doses (18). AI is also beginning to play a prominent role in emerging areas such as immunotherapy and nanomedicine, where advanced algorithms help design nanoparticles or predict the immune response to specific therapies (19). These applications are not merely theoretical: in well-equipped healthcare systems, AI-based tools have already been rapidly implemented clinically.

Although this adoption is not occurring uniformly worldwide, there are reasons for optimism. AI can be a powerful tool for reducing global gaps (20), for example, through clinical decision support systems in regions with a shortage of oncologists or through automated diagnoses where the number of specialists is limited (4, 21). In contexts where maximum efficiency with minimal resources is required, AI could help democratize access to international standards of care (22).

The current landscape of profound technological change is not only transforming the clinical environment but also posing significant challenges for medical education (15). As AI becomes integrated into the oncology workflow (23), it is essential that new generations of healthcare professionals understand how to use these tools effectively and ethically (24), including the ability to understand AI's capabilities and limitations (15), correctly interpret its results (15), and make informed clinical decisions in collaboration with intelligent systems (25). AI literacy should not be considered an optional technical skill but a fundamental competency in 21st-century medicine (26). Therefore, integrating AI content into medical and oncology curricula is already considered an urgent priority (27).

In this moment's educators recognize that familiarity with these technologies will be essential for current and future medical practice (28). Additionally, curriculum design must not only include technical aspects (models, algorithms, accuracy) but also address the ethical, legal, and social dimensions of AI use in healthcare, such as algorithmic bias, data privacy, and clinical responsibility. A comprehensive educational approach will enable the use of AI not only as a technical tool, but also for critical reflection on its systemic impact on cancer care (29).

This study explores research describing how AI has been implemented in cancer education in diverse global contexts, evaluating the clinical and educational integration of these tools. It identifies patterns, challenges, and opportunities that can guide future strategies for incorporating AI effectively, equitably, and ethically into oncology between 2019 and 2024.

## 2 Methodology

This systematic review employed a rigorous, structured, and transparent methodological framework to identify, evaluate, and synthesize the academic literature focused on the implementation of AI in cancer-related medical education, with a special emphasis on its clinical and pedagogical integration in diverse global contexts. The methodological approach was guided by the principles established in systematic review protocols, incorporating key elements of the PRISMA guidelines (Preferred Reporting Items for Systematic Reviews and Meta-Analyses) to ensure replicability, clarity, and comprehensiveness (30, 31). The main objective of this study was to analyze empirical studies that reported on the design, implementation, and evaluation of educational interventions integrating AI into oncology training for healthcare professionals. This included interventions in undergraduate, postgraduate, and continuing medical education that documented measurable educational outcomes, such as knowledge acquisition, diagnostic accuracy, clinical reasoning, skills development, and student engagement with or acceptance of AI technologies in oncology contexts. The literature search was conducted in multiple academic databases, including Scopus, PubMed, and IEEE Xplore.

### 2.1 Design review, application of the PRISMA and PICO methodologies

The methodological rigor, transparency, and reproducibility of this systematic review were ensured through the PRISMA guidelines (Preferred Reporting Items for Systematic Reviews and Meta-Analyses), and the PICO framework (Population, Intervention, Comparison, Outcome) served as a guiding framework. These established methodological standards enabled a structured and comprehensive approach to identifying, selecting, and synthesizing relevant literature on integrating AI into cancer-related medical education, with a focus on both clinical applications and pedagogical strategies in diverse global contexts.

Using the PICO model, the research question was iteratively refined to align with the objectives of this review: to explore the educational impact of AI technologies on medical training in oncology. The final research question that guided this review was:

“*How has AI been implemented in cancer-related educational programs for students and healthcare professionals in general?”*

Based on this question and the PICO framework (Table 1), a structured Boolean query was developed to retrieve relevant literature using explicit Boolean grouping of the artificial intelligence, education/training, and oncology concept blocks. The approach was applied in Scopus, PubMed, and IEEE Xplore for 2019–2024; full queries and counts appear in Table 2.

**Table 1:** PICO framework describing the core elements used to guide the research question and inclusion criteria for this systematic review on the implementation of AI in cancer-related medical education.

	Element	Description
P	Population	Medical students, residents, oncology trainees, and healthcare personnel involved in cancer-related medical education
I	Intervention	Educational programs or strategies that integrate AI technologies, specifically in teaching and training about cancer.
C	Comparison	Traditional or non-AI-based educational methods
O	Outcome	Measurable educational outcomes such as: knowledge acquisition, skills development, cognitive performance, diagnostic accuracy.

**Table 2:** Database search results using structured Boolean queries focused on the intersection of AI and oncology education, in the range 2019–2024.

Database	Query	Results
Scopus	"Artificial intelligence” AND “Education” OR “Training” AND “Cancer” AND “Medical"	561
PubMed		443
IEEE Xplore		390

### 2.2 Database selection and search strategy

To ensure a comprehensive and multidisciplinary collection of articles, three major academic databases were selected for this review: SCOPUS, PubMed, and IEEE Xplore. Each database contributes uniquely to the intersection of healthcare, education, and emerging technologies, making them essential for capturing diverse research perspectives.

SCOPUS was selected for its extensive indexing of peer-reviewed journals in health sciences, education, and engineering.
PubMed was selected for its rigorous selection of biomedical literature, including clinical and educational research specific to healthcare training.
IEEE Xplore was included to capture technical and engineering contributions to AI and simulation technologies, particularly those not indexed in traditional medical databases.

The Boolean formulation of the main keywords, focused on the topic (Table 2), aims to encompass research at the intersection of AI technologies and immersive educational methods in oncology. The study used the period from 2019 to 2024, a time of exponential advances in Generative AI, relevant to adaptive learning and intelligent tutoring systems. This was followed by the catalytic digital transformation brought about by the COVID-19 pandemic (2020–2022) regarding the style and environment of medical education practice. This ensures the inclusion of the most up-to-date and relevant literature, encompassing both early adoption studies and recent advances in the field.

The search strategy was based on a concise yet highly specific Boolean query. Given the high volume of AI-related publications in recent years, particularly in general healthcare settings, broader queries risked retrieving a large number of non-educational and non-oncological articles. Limiting the search to this central intersection allowed us to:

Prioritize articles with explicit pedagogical or curricular content related to AI and oncology;
Minimize irrelevant matches from overlapping fields, such as general radiology, pure algorithmic development, or unrelated disease models;
Preserve the systematic integrity of the selection and eligibility process by selecting studies most aligned with the review's PICO framework.

**Figure 1:**
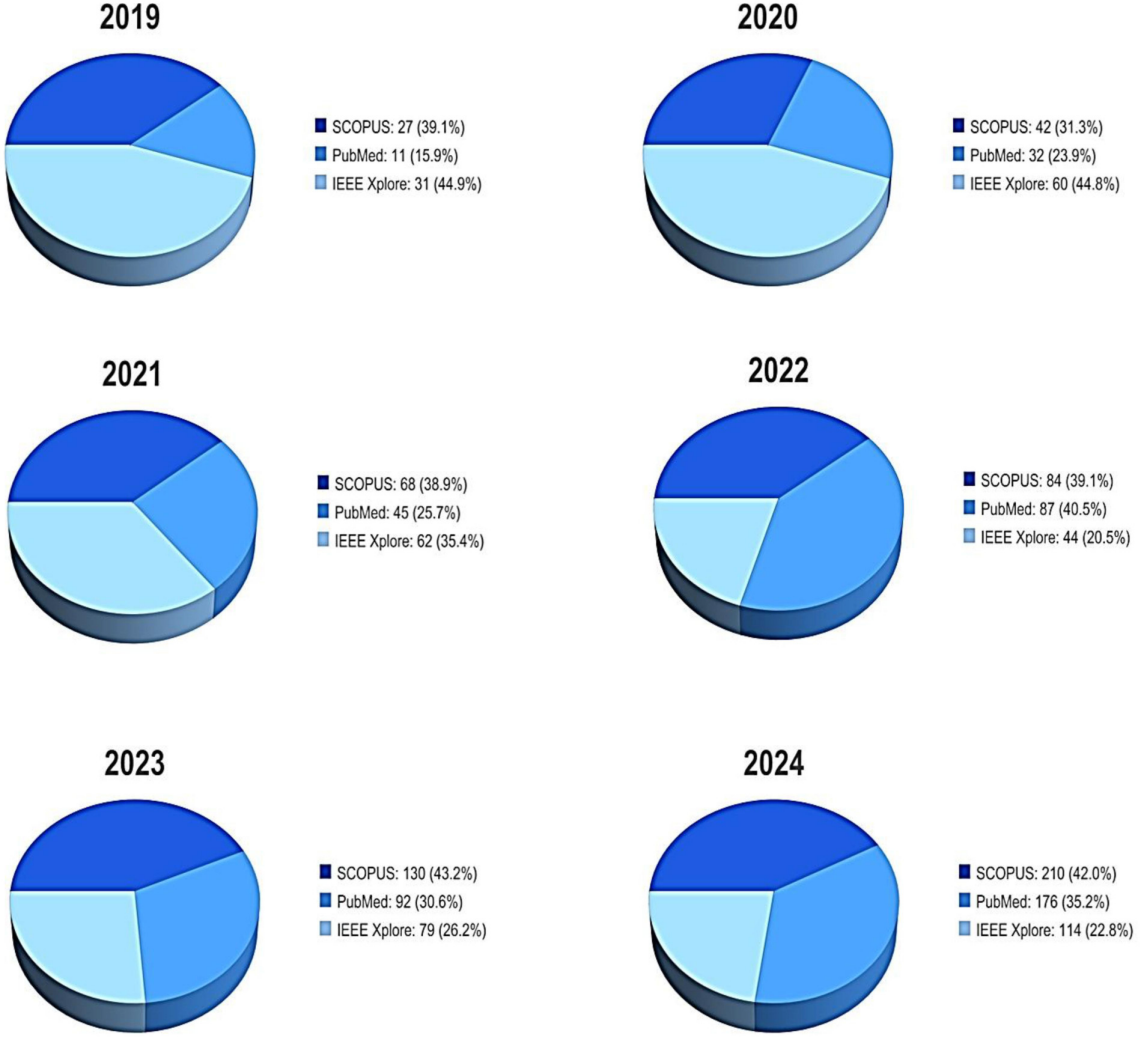
Annual distribution of records retrieved from Scopus, PubMed, and IEEE Xplore (2019–2024).

The volume and evolution of the retrieved publications reveal trends that allow for a better understanding of the evolution of interest in AI research in cancer-focused medical training during the period (2019–2024). As detailed in Figure 1, publication output is observed across all databases, with Scopus consistently registering the highest number of annual contributions, followed by PubMed and IEEE Xplore. A progressive increase in the total volume of publications is evident, with notable growth in contributions to Scopus and PubMed in 2023 and 2024, reflecting the growing interest and research activity in AI-driven oncology training across all medical and technical disciplines.

### 2.3 Identification

**Figure 2:**
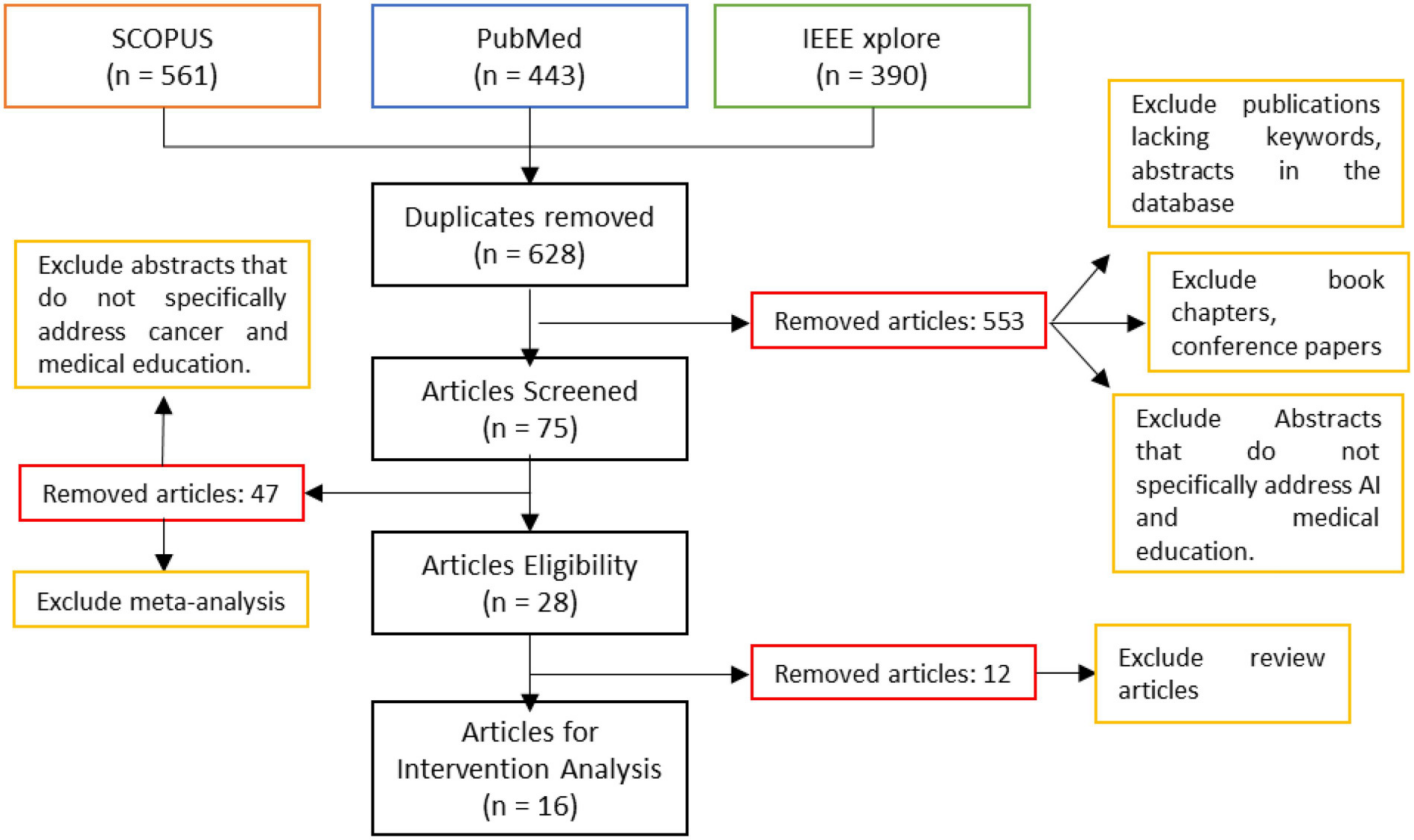
PRISMA flowchart showing the selection process of studies across four phases: identification, screening, eligibility, and inclusion.

The process of identifying and selecting studies for this systematic review was carried out in accordance with the PRISMA principles. Figure 2 illustrates in detail the different phases of the process, from initial identification to final inclusion of articles for analysis.

The initial identification phase focused on conducting a comprehensive and reproducible literature search. The primary database selected for this review was Scopus, due to its broad coverage of high-quality scientific literature in the fields of health sciences, engineering, and technology. The initial Scopus search, limited to records dated 2019–2024, yielded a total of 561 records (*n* = 561). These records represented the raw dataset potentially relevant to our research question: the implementation of AI in oncology, with a focus on educational and clinical integration.

In parallel, and to ensure the capture of fundamental technical literature in the area of AI, a complementary search was conducted in IEEE Xplore, a key database for literature in electrical engineering, electronics, computer science, and related fields. From this database, 390 records were identified (*n* = 390). It is important to note that, of the total records identified in Scopus (561), a subset of 443 articles had already been published in peer-reviewed journals, while the remainder consisted of pre-publication articles or articles in other stages of publication. The combined set of records identified through both databases was 951 records (561 + 390).

### 2.4 Screened

Database searches yielded 951 records. After deduplication (553 duplicates removed), 398 unique records were screened by title/abstract against predefined criteria. Of these, 323 were excluded at screening, and 75 reports were retrieved for full-text assessment.

### 2.5 Eligibility

Seventy-five full-text articles were assessed for eligibility. A more stringent set of criteria was applied to ensure dual relevance to oncology and medical education with an explicit AI component. Articles were excluded for prespecified reasons: (i) not addressing AI within a cancer-education context (cancer and AI and education), (ii) review, meta-analysis, protocol, opinion piece, book chapter, conference paper, or record lacking abstract/keywords, (iii) no learning outcomes, or (iv) duplicate/overlapping dataset. Fifty-nine full-text reports were excluded for these reasons. After full-text assessment, 16 studies were included in the qualitative synthesis (Figure 2). Study quality was appraised using a prespecified, structured process applied uniformly across included studies, and the resulting judgments informed the synthesis.

### 2.6 Inclusion

Sixteen studies were included in the qualitative synthesis (Figure 2). These studies constitute the dataset for the intervention analysis reported in Section 3.

## 3 Results

The final analysis focused on 16 studies describing specific AI interventions or applications in oncology education and training. This final sample, though small, reflects an emerging and highly specialized body of literature encompassing theoretical concepts, practical implementation, training, and clinical practice in oncology, and ranging from educational tools for medical students to decision support systems for experienced oncologists. Analysis of these studies reveals their distribution into two main, interconnected categories: (1) Educational Integration, where AI acts as a pedagogical tool to enhance understanding and skills; and (2) Clinical Integration, where the focus is on the practical application of AI to assist in diagnosis, treatment planning, and precision oncology.

### 3.1 AI technologies in cancer education: trends in pedagogical utility, efficacy, and limitations

Table 3 shows that AI applications in cancer education have expanded significantly in recent years, reflecting growing interest in improving medical training, patient engagement, and clinical readiness. One prominent trend is the widespread use of AI-based chatbots and conversational agents, particularly those based on large language models. These systems have been applied in both patient education and professional training, often aiming to improve information delivery, decision support, and engagement in cancer-related situations. In this case (32), and (33) explored the use of the AI chatbot PROSCA for prostate cancer education. Both studies integrated natural language processing (NLP)-based chatbot platforms as complementary tools to standard information channels. The technology was primarily implemented in clinical settings to help patients or students understand the concepts of early detection and cancer screening.

**Table 3:** Summary of empirical studies implementing AI technologies in cancer-related education: interventions, measured variables, observed effects, limitations, and types of AI tools.

Authors	Intervention	Variable	Effect	Limitations	Technology type
Baumgärtner et al. (32)	PROSCA AI chatbot as a complement to standard information on prostate cancer	Engagement	Positive	Moderate sample size; single-center study; very specific clinical application	AI/NLP-based medical chatbot
Kinoshita et al. (39)	AI model for nerve recognition in laparoscopic/robotic rectal surgery, used in educational videos	Skill development	Positive	Small sample; single center; only 60 test frames; preliminary results	U-Net–type neural network for segmentation of nerve structures
Roldan-Vasquez et al. (34)	Evaluation of ChatGPT responses to American Society of Breast Surgeons FAQs on breast surgery	Performance	Increased	Limited to 9 questions; dependent on a specific ChatGPT version; no direct patient perspective	Public LLM-based chatbot (ChatGPT)
Lee et al. (35)	Use of ChatGPT to generate preoperative counseling material for five oncologic head and neck surgeries	Performance	Increased	Only five surgery types; evaluators from a single center; no patient involvement	LLM-based chatbot (ChatGPT)
Monteiro Cordeiro et al. (42)	Creation of an open mammography (2D and 3D) database with advanced search system for AI and teaching	Engagement	Positive	Relatively small number of cases; single-center bias; no large-scale educational validation yet	Mammography image database with structured search engine
Atarere et al. (37)	Evaluation of online chat models (ChatGPT, YouChat, BingChat) for education on colorectal cancer screening	Performance	Increased	Limited to 20 questions (15 key concepts+5 common questions); no patient participation	General-purpose AI chatbots (LLMs)
Wang et al. (41)	“ThyroAIGuide” platform with GPT-4 to analyze thyroid ultrasound reports and suggest diagnosis and plan; interpretability assessed with Chain-of-Thought	Performance	Increased	Single-center data; 109 cases; diagnostic performance still insufficient for autonomous use	GPT-4 LLM with Chain-of-Thought reporting for imaging
Odabashian et al. (36)	Use of ChatGPT-3.5 to answer >1,000 ASCO-SEP question-bank items; performance compared with correct answers	Performance	Increased	Only one model/version evaluated; no simulation of real clinical scenarios; no human comparison group	LLM-based chatbot (ChatGPT-3.5)
Hesso et al. (47)	Survey and interviews with professionals on AI in cancer care (INCISIVE H2020); needs and training in AI explored	Engagement	Increased	Relatively small sample (95 surveys, 27 interviews); multiple countries but not representative	AI technologies for medical imaging (no single platform)
Tomioka et al. (40)	AI system to highlight hepatic veins and Glissonian pedicles in laparoscopic hepatectomy; also used as an educational tool	Performance	Positive	Annotation of only 350 frames; single center; no data on clinical outcome impact	Deep neural network for segmentation of tubular structures
Pan et al. (38)	Comparison of 4 chatbots (ChatGPT 3.5, Perplexity, Chatsonic, Bing AI) answering top Google queries for 5 common cancers	Performance	Positive	Only 5 tumor types and 5 queries per tumor; no real patient feedback	LLM-based chatbots (4 platforms)
Catanuto et al. (43)	Use of Word2Vec to transform breast cancer textbooks into a vector corpus and explore semantic similarities	Performance	Positive	Corpus limited to 6 books; no evaluation of actual learning impact	Word2Vec neural network/semantic embeddings
Görtz et al. (33)	Development and initial evaluation of the PROSCA chatbot for education on early detection of prostate cancer	Engagement	Positive	Very small sample (10 patients); no control group; exploratory results	AI/NLP-based medical chatbot (PROSCA)
Heald et al. (44)	AI chatbot to apply CCRAT, educate on hereditary CRC syndromes, and obtain consent for genetic testing during colonoscopy preparation	Engagement	Positive	Not all invitees participated; single-center study; no detailed subjective experience evaluation	AI-based chatbot with CCRAT questionnaire and educational module
Chavez-Yenter et al. (45)	Automated conversational agent delivering pretest genetic education to primary care patients eligible for cancer risk evaluation	Engagement	Positive	Small sample (30 complete the chat); no control group; potential selection bias	Automated mobile/web conversational agent (chatbot)
Gillan et al. (46)	Focus groups with radiation medicine professionals on professional implications of introducing AI	Engagement	Increased	24 participants from a single center; qualitative design; limited generalizability	AI-assisted treatment planning systems

Other studies used public or private AI-oriented language models to generate or evaluate educational content about cancer (34). and (35) evaluated the performance of ChatGPT in answering frequently asked questions or generating materials for patients in oncological surgery. These studies highlight how generative AI is being tested in clinical communication, preoperative counseling, and health literacy (36). in their study highlights the expansion of this trend by using ChatGPT-3.5 to answer more than 1,000 questions from an oncology forum, representing a direct application of AI modeling guidance in medical exam preparation and the reinforcement of theoretical knowledge.

Focusing on comparative evaluations of multiple AI-powered chat platforms. For example (37), evaluated three models (ChatGPT, BingChat, YouChat) in the context of colorectal cancer screening education, while (38) compared four AI-based language modeling chatbots focused on answering common, publicly available cancer-related queries. The results reflect the proliferation of general-purpose chatbots in health education and underscore the need for rigorous validation of the quality, consistency, and clinical relevance of their content.

In another area focused on implementing AI technologies centered on medical imaging or surgical support, the research by (39) introduced a U-Net-based neural network designed for nerve recognition in laparoscopic rectal surgery, which was integrated into educational video modules for students and residents (40). In their research they managed to develop a deep learning segmentation tool that highlights the hepatic veins and Glissonian pedicles during hepatectomy, used as real-time visual support for training in surgical anatomy (41) developed the “ThyroAIGuide” platform, which combined GPT-4 and thought chain reasoning to analyze thyroid ultrasound reports and generate diagnostic and treatment suggestions. Although in an early validation phase, this system represents a sophisticated convergence of an AI-oriented language model and radiological decision support tools, with potential implications for both clinical workflow and educational feedback mechanisms. Similarly (42), proposed a unique approach by creating an open-access mammography database with 2D and 3D images linked to a structured search system. While this approach is not a machine learning tool itself, the resource was explicitly designed to facilitate both AI training and human instruction, bridging the gap between dataset availability and educational utility in breast cancer diagnosis. While (43) applied Word2Vec embeddings to breast cancer textbooks to examine semantic associations and content structure, representing an innovative application of natural language understanding for curriculum improvement in medical education, primarily focused on cancer.

Beyond tools aimed at medical professionals, several studies focused on patient-facing AI-based platforms that facilitate education and consent for genetic testing. For example (44), developed a chatbot that uses the Colorectal Cancer Risk Assessment Tool (CCRAT) to inform patients about hereditary syndromes and facilitate consent for genetic testing (45). implemented an automated conversational agent to deliver pre-test genetic education in primary care. In contrast (46), conducted focus groups with radiology professionals to explore the broader implications of AI for competency development, workflows, and future training needs.

Despite the promising nature of these technologies, methodological limitations were common across all studies. Most reported small sample sizes, single-center recruitment, and the absence of control groups, which limited the generalizability and robustness of the findings. For example (32), and (33) conducted exploratory studies with a limited number of participants and without a comparative design. Similarly (35), and (34) evaluated only a limited range of oncological procedures or questions, raising concerns about the comprehensiveness and future scalability of their approaches.

**Figure 3:**
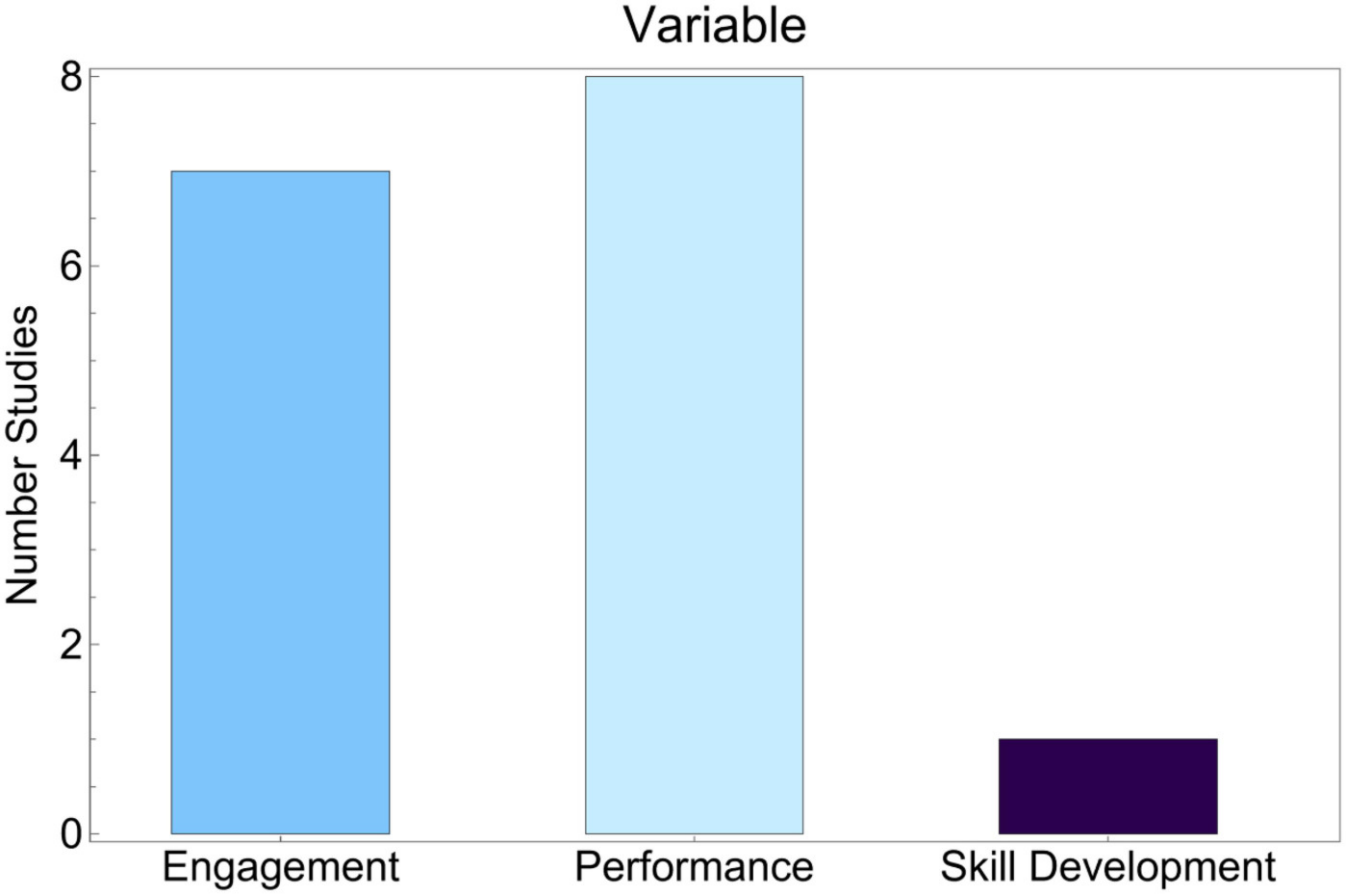
Distribution of educational variables targeted in AI-driven cancer education studies. Bars indicate the number of included studies per category (*y*-axis=number of studies).

Figure 3 summarizes the distribution of educational variables assessed in the selected studies. Three categories were identified, performance, engagement, and skills development. Based on the number of studies in each category, the evidence base focuses more often on cognitive and information-processing outcomes than on procedural or technical skills. Performance outcomes commonly captured comprehension, information quality, response accuracy, or decision-related task performance when interacting with AI-generated content, particularly chatbots. Studies (34–37, 40) and (43) evaluated performance-oriented endpoints in educational contexts, while selected clinical integration studies with performance testing components were categorized separately in Table 4.

**Table 4:** Summary of selected studies that evaluate the integration of AI in cancer education in clinical and educational domains.

Authors	Area	Stage of employed technology	Number of participants	Country	Primary intent (E/C)^a^
Baumgärtner et al. (32)	Prostate cancer patient education/diagnostic counseling	Clinical prototype already deployed and evaluated in an RCT	112 partipants	Germany	E
Kinoshita et al. (39)	Surgical training in colorectal cancer	Research prototype, offline video analysis; proof of concept	5 colorectal surgeons for main survey	Japan	E
Roldan-Vasquez et al. (34)	Patient education in oncologic breast surgery	Publicly available tool evaluated in a research setting	4 breast surgeons as evaluators	USA	E
Lee et al. (35)	Preoperative education in head and neck cancer surgery	Use of ChatGPT as an external tool, without formal clinical integration	5 head and neck surgeons	USA	E
Monteiro Cordeiro et al. (42)	Educational resources in breast imaging/breast cancer	Functional online platform in use for research and teaching	100 patient cases; number of end users NR	Brazil	E
Atarere et al. (37)	Patient education on colorectal cancer screening	Public tools already available, evaluated in a research setting	Clinical evaluators	USA	E
Wang et al. (41)	Decision support for thyroid cancer and potential educational tool for clinicians and patients	Advanced prototype evaluated retrospectively; online test platform	109 thyroid cancer cases; multiple physicians at different levels	China	C
Odabashian et al. (36)	Education and self-assessment in medical oncology	Public tool evaluated outside clinical practice	1,040 question-bank items; no patients involved	USA	E
Hesso et al. (47)	Professional training and AI adoption in oncologic imaging (breast, lung, colon, prostate)	AI still at design/project stage (INCISIVE), not widely deployed	95 HCPs in survey; 27 in interviews	Europe	E
Tomioka et al. (40)	Surgical education in hepatic cancer (hepatectomy context)	Real-time prototype tested in OR and educational sessions	10 hepatobiliary surgeons; 10 students/residents for educational evaluation	Japan	E
Pan et al. (38)	Public cancer information (skin, lung, breast, colon, prostate)	Already-available chatbots, used outside clinical context	100 responses	USA	E
Catanuto et al. (43)	Theoretical education in breast cancer (for students/professionals)	Research phase; text-mining tool without clinical deployment	No reported	Europe	E
Görtz et al. (33)	Patient education on early detection of prostate cancer	Early chatbot prototype, pilot testing	10 men with suspected prostate cancer	Germany	E
Heald et al. (44)	Education and genetic screening in hereditary colorectal cancer	Functional chatbot deployed in clinical practice as an extension of genetic counseling	4,254 invited	USA	C
Chavez-Yenter et al. (45)	Pretest genetic education for cancer in primary care	Pilot conversational-agent platform, early research/implementation phase	103 invited	USA	E
Gillan et al. (46)	Professional training and roles in radiation oncology	AI-assisted treatment planning in early implementation phase	24 professionals	Canada	C

^a^ E, Educational integration; C, Clinical integration (clinical decision support).

In contrast, studies focused on engagement often involved user interaction with AI tools in educational or counseling contexts, particularly through conversational agents (32, 33, 42, 44, 47). and (46) evaluated chatbots used to educate patients about cancer screening or genetic testing. These interventions explored aspects such as user satisfaction, willingness to reuse the tool, and completion rates for educational modules or informed consent processes. Notably, many of these studies involved chatbots based on Natural Language Processing (NLP) or mobile/web conversational platforms, resulting in improved access to and user experience in patient-centered education.

Only one study (39),, specifically addressed skills development. This intervention employed a U-Net-based neural network for nerve recognition in laparoscopic surgery and was integrated into educational videos used by residents. The limited representation of procedural skills in the literature indicates a possible gap in current research: AI applications in practical and technique-oriented training in oncology remain largely unexplored.

**Figure 4:**
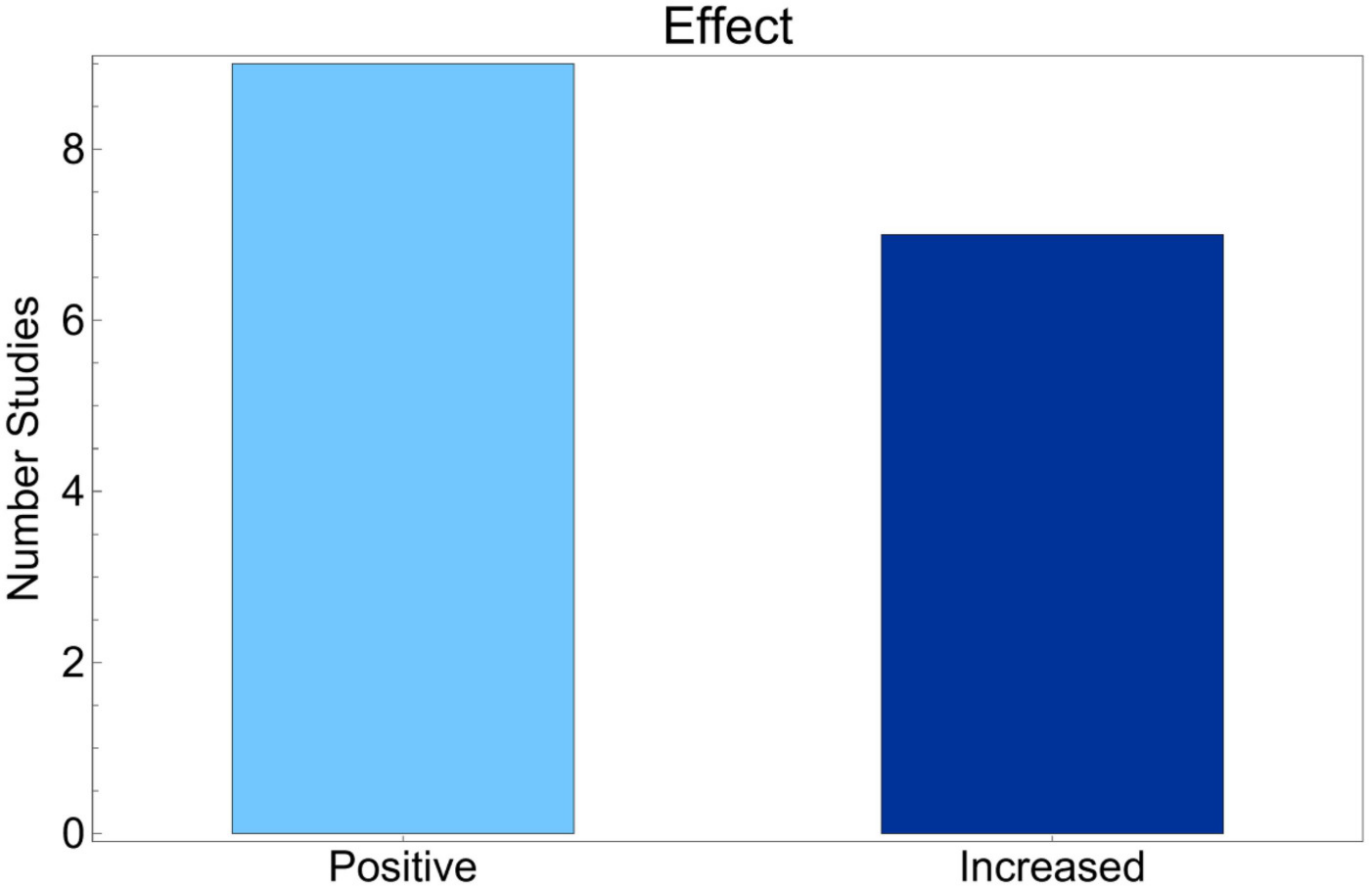
Distribution of reported effects in AI-based educational interventions for cancer education.

As summarized in Figure 4, most included studies reported favorable outcomes after the use of AI tools, categorized as positive or increased. Here, positive denotes favorable findings based primarily on subjective or qualitative evaluations, whereas increased denotes improvement reported in quantitative outcome metrics; these labels summarize reporting type and direction and are not used to imply effect size magnitude. Across studies coded as positive, outcomes primarily reflected perceived benefit and acceptability (e.g., satisfaction, usability, willingness to reuse). Studies coded as increased more often reported improvements in quantitative metrics, but these were typically short-term and heterogeneous, limiting inference about sustained educational effectiveness. Nine studies were coded as positive and seven as increased, and no included study reported neutral or adverse educational outcomes.

The distinction between positive and increased outcomes is relevant for interpretation. Studies coded as positive most often relied on subjective or qualitative endpoints such as user satisfaction, perceived usefulness, or acceptability. For example, the PROSCA prostate cancer chatbot studies (32, 33) reported favorable reception and willingness to reuse the tool, primarily based on post-intervention surveys and interviews. Similarly, the mammography training platform described in (42) received positive feedback from early users, supporting feasibility and acceptability without necessarily demonstrating measurable learning gains.

Studies coded as increased more frequently reported quantitative improvements in performance-oriented metrics such as accuracy, test scores, or comprehension. Odabashian et al. (36) evaluated ChatGPT-3.5 on the ASCO-SEP question bank, reporting 56.1% overall accuracy with variation across domains, which supports its potential role as a scalable tool for knowledge review while remaining below typical examination thresholds.

One of the most prominent types of technology observed in the included studies was AI-based language model chatbots, including ChatGPT and other conversational agents. These tools have become increasingly popular in both face-to-face and professional learning environments (35). used ChatGPT to generate preoperative counseling material for cancer surgeries and observed that participants rated it similarly to conventional health websites; likewise (37), compared multiple AI platforms to assess their accuracy in colorectal cancer screening education (38). conducted a broader comparison of four chatbots for five cancer types, also reporting high information quality.

Another group of studies applied AI to support decision-making and the interpretation of medical images, often combining educational objectives with tools for clinical decision-making. In this case, study (41) introduced “ThyroAIGuide,” a GPT-4 platform with chain-of-thought reasoning designed to analyze thyroid ultrasound reports and recommend diagnoses. Although its diagnostic accuracy remained limited, the platform was reported to improve report clarity and user interpretability (40). and (39) employed a tool that also implemented neural network-based tools for the recognition of anatomical structures in surgical procedures, which were subsequently repurposed for resident training.

Despite the generally favorable findings, the reviewed studies also reveal several persistent methodological limitations. A recurring limitation in almost all studies was the small sample size, as many were single-institution pilot or feasibility projects (32, 45). reported the results of a small cohort of users at a single center. These limited population samples restrict the external validity of the findings, especially across different educational systems, cancer types, and levels of digital literacy.

Very few studies implemented control groups, randomized designs, or longitudinal follow-up to assess knowledge retention or behavioral change over time. While increased use of AI tools appears beneficial in the short term, it remains unclear whether this translates into sustained learning improvements or better clinical outcomes (39)., for example, documented improvements in neural recognition after exposure to AI-powered educational videos, but did not provide post-intervention follow-up or a comparison with traditional teaching methods.

**Figure 5:**
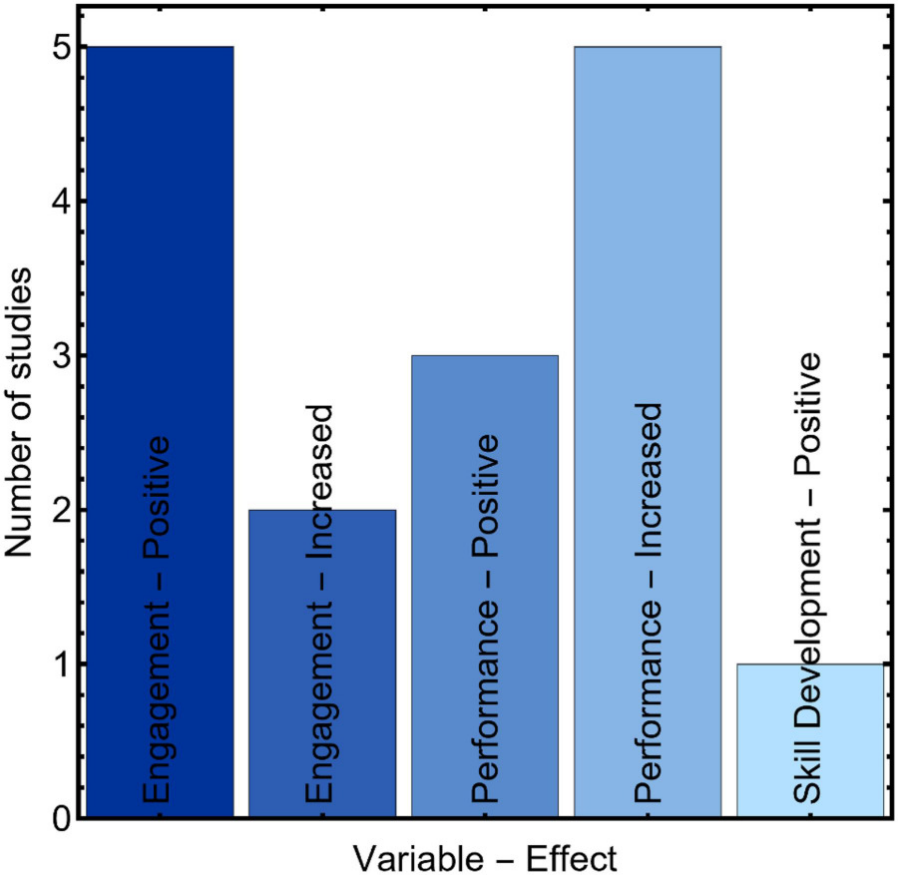
Reported effects of AI implementation in oncology education contexts.

Figure 5 summarizes the reported outcome categories by domain, combining engagement, performance, and skills development. Engagement outcomes were commonly coded as positive or increased. Positive engagement findings were reported in studies evaluating prostate cancer chatbots such as PROSCA (32, 33), where users described high receptiveness and willingness to interact with the tool. Increased engagement was also reported in studies involving exploratory learning interfaces, including a mammography image database that encouraged active navigation of clinical content (42). Engagement-related findings were additionally described in studies focused on implementation and workforce readiness, including genetic counseling workflow chatbots and professional role adaptation, where interactivity and user acceptance were emphasized (44–47). These engagement outcomes were largely based on subjective evaluations of usability, satisfaction, or perceived usefulness.

Performance was the most frequently assessed domain and included both positive and increased categories in Figure 5. Performance-oriented outcomes were typically evaluated through accuracy, content quality, comprehension, or structured ratings of AI-generated information. ChatGPT-based evaluations in breast surgery education and preoperative counseling reported favorable expert ratings for clarity and completeness (34, 35), while screening education studies comparing chatbot outputs against guideline expectations reported generally acceptable performance with identified limitations (37). Odabashian et al. (36) evaluated ChatGPT-3.5 on the ASCO-SEP question bank and reported 56.1% overall accuracy, illustrating moderate knowledge coverage relevant to educational use. A smaller subset of studies addressed performance in tools primarily oriented toward clinical decision support, which were classified as clinical integration in Table 4 and interpreted accordingly in this review (41, 44, 46).

Skills development was infrequently assessed. Only one study reported a skills-related outcome, describing improved recognition of nerve structures in robotic rectal surgery using a segmentation model (39). This pattern suggests that current AI applications in oncology education predominantly target cognitive outcomes, while procedural or psychomotor skill development remains underrepresented in the empirical literature.

### 3.2 Evolving technologies and global implementation of AI in cancer education settings

**Figure 6:**
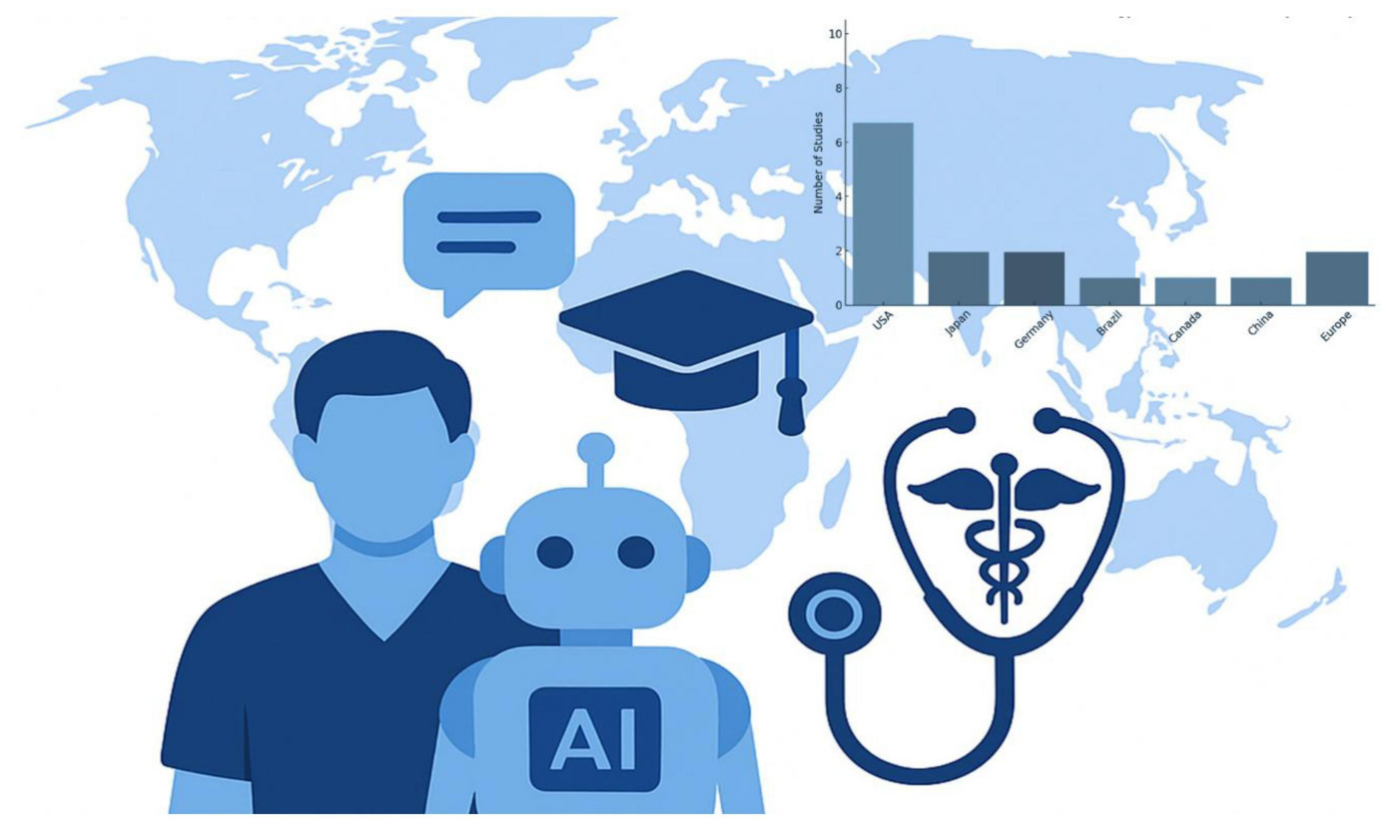
Global overview of AI implementation in cancer education.

Figure 6 illustrates the international distribution of studies on AI-based educational tools in oncology, highlighting the predominance of contributions from the United States. The integrated bar chart shows the number of studies identified by country, with the US leading, followed by Japan and Germany. Technologies ranged from fully implemented clinical prototypes to investigational chatbots and text mining models. Participants included patients, clinicians, educators, and students, with sample sizes ranging from small pilot groups to thousands of users in real-world implementations. The majority of studies originated in the United States, followed by Japan and Germany, reflecting global but uneven research activity in AI-enhanced cancer education.

Table 4 summarizes how AI applications in oncology are developing across regions and subfields and includes a Primary intent label to distinguish educational integration from Clinical integration. This distinction is used to avoid treating clinical decision support systems as educational interventions when education is not the main objective.

AI chatbots for patient education and counseling were a prominent Educational integration application. In Germany, Baumgärtner et al. (32) developed PROSCA for prostate cancer counseling and evaluated it in an RCT with 112 men with suspected prostate cancer, reporting reduced unmet information needs and improved understanding compared with standard care. An earlier German pilot (33) tested a preliminary chatbot version for early detection education in 10 men, showing high usability and self-reported knowledge gains, and supporting progression from feasibility testing to controlled evaluation.

In the United States, ChatGPT was assessed as a counseling resource in several contexts. Lee et al. (35) used ChatGPT to generate preoperative education content for head and neck cancer procedures and five surgeons judged outputs comparable to standard online information, with preference for AI-generated versions in nearly half of cases. Roldan-Vasquez et al. (34) evaluated responses to breast surgical oncology FAQs and four breast surgeons rated clarity and practicality highly, with reliability averaging about 4 out of 5. Atarere et al. (37) compared ChatGPT, Bing Chat, and YouChat for colorectal cancer screening concepts and FAQs and found responses generally acceptable but with occasional inaccuracies and limitations in updated recommendations, supporting cautious use and clinician oversight.

Clinical integration was illustrated by studies in which the primary aim was pathway support or decision support, while educational content was secondary. Wang et al. (41) evaluated a thyroid cancer decision support prototype using clinical cases in an online testing setting. Heald et al. (44) reported a chatbot integrated into a US healthcare system for hereditary colorectal cancer risk assessment and genetic counseling workflows, where 4,254 patients were invited, about 506 initiated the chat and 487 completed it, and the system supported triage and embedded pre-test education, with 129 patients proceeding to multigene panel testing and pathogenic variants identified in about 9 percent of those evaluated. Chavez-Yenter et al. (45) described BRIDGE for pre-test genetic education in primary care, where 103 patients were invited, 36 initiated the chat and 30 completed it, and 70 percent of completers opted to proceed with genetic testing after the session.

Outside formal care pathways, Pan et al. (38) evaluated responses from publicly available chatbots to common online cancer queries and reported high information quality with a mean DISCERN score (a validated instrument for assessing the quality of consumer health information) of 5 out of 5 and no overt misinformation, while readability and usability remained concerns, with college-level reading levels, PEMAT comprehension around 67 percent, and actionability around 20 percent.

Educational integration also included skills training and knowledge development. In Japan, Kinoshita et al. (39) described an early-stage system that analyzed laparoscopic colorectal surgery videos to recognize key structures, with potential value for feedback in training. Tomioka et al. (40) evaluated an intraoperative guidance system for laparoscopic hepatectomy, reporting moderate segmentation performance and perceived educational usefulness among trainees observing AI-augmented procedures. In Brazil, Monteiro Cordeiro et al. (42) developed an online mammography platform with 100 anonymized cases and more than 900 images to support teaching and research. Odabashian et al. (36) assessed ChatGPT-3.5 on 1,040 ASCO-SEP questions, with overall accuracy around 56.1 percent, reaching 80 percent in developmental therapeutics and about 49 percent in gastrointestinal cancers, indicating moderate coverage that does not replace formal training.

Professional readiness and role adaptation were examined in Europe and Canada. INCISIVE (47) surveyed 95 healthcare professionals across six European countries and interviewed 27, finding generally favorable attitudes toward AI but persistent concerns about training, workflow integration, and role changes. Gillan et al. (46) explored early implementation of AI-assisted treatment planning in radiation oncology and reported expected task redistribution and new training needs for interpreting AI outputs and maintaining complementary skills, consistent with a primary Clinical integration focus.

Overall, the Primary intent labeling clarifies how outcomes should be interpreted across studies. Learning-focused work mainly reports feasibility, perceived usefulness, and short-term educational endpoints, while clinically integrated tools emphasize implementation, workflow, and safety, with educational effects typically secondary (32–46).

### 3.3 Implementation barriers and evaluation characteristics of AI interventions

The selected studies shown in Table 5, which span diverse global contexts, from tertiary hospitals and specialist clinics to academic training programs and international surveys, highlight common barriers such as technical integration problems, a lack of specific AI training, and concerns about data accuracy. Integrating AI tools into existing clinical workflows has proven difficult in practice. In a randomized trial in a urology service, an AI system was used during the diagnosis of suspected prostate cancer, and researchers noted difficulties integrating the tool into clinical systems and the need to personalize AI results for different patient profiles (32). Similarly, a pilot implementation in a hospital urology clinic revealed uncertainty about the additional benefits of AI and potential selection bias, underscoring the difficulty of incorporating AI into routine practice without disrupting existing processes (33). In surgical oncology settings, researchers found that AI adoption must be aligned with clinical workflow and technical constraints. A study in a surgical training setting described how an AI-assisted system for intraoperative guidance faced workflow integration challenges and technical validation needs, including concerns about over- and under-detection of surgical targets and dependence on input video quality for nerve recognition accuracy (39). Another study, focusing on a laparoscopic hepatectomy training program, observed technical recognition errors by the AI and emphasized the need for rigorous validation of AI outputs before surgeons and residents can rely on and effectively use the technology (40). These findings across different clinical scenarios demonstrate that technical and workflow integration challenges are a recurring theme: ensuring that AI tools function reliably within established care pathways and technical environments is often easier said than done.

**Table 5:** Summary of barriers, study designs, duration of interventions, assessment tools, training procedures, and clinical or educational contexts in the included studies that evaluate AI-based systems in oncology training and care pathways.

Authors	Barriers/challenges	Study design	Duration of intervention	Assessment tools	Training provided	Context/setting
Baumgärtner et al. (32)	Integration into clinical systems; need to personalize responses for different patient profiles	Randomized controlled trial	Use during the diagnostic workup	Study-specific questionnaires on information need, use, and satisfaction	No formal training	University hospital urology service, care for suspected prostate cancer
Kinoshita et al. (39)	Need for integration into intraoperative workflow; risk of under-/overdetection; dependence on video quality	Technical validation study	Exposure (1 min of AI video)	Dice/IoU metrics; questionnaires on utility and under-/overdetection; 12-point nerve recognition test	Brief explanation of the system before viewing videos	Tertiary colorectal surgery center, educational sessions and questionnaires
Roldan-Vasquez et al. (34)	Potential risk of outdated content or small errors; need for clinical oversight	Cross-sectional study	Single round of answer generation and evaluation	Likert-scale reliability (1–5); Patient Education Materials Assessment Tool (PEMAT)	No formal training	Academic evaluation of AI online content about breast cancer
Lee et al. (35)	Dependence on automatically generated content; concern about potential medical errors	Cross-sectional study	Single generation and evaluation per procedure	Readability, completeness, accuracy, and error counts; preference choice	No formal training	Tertiary head and neck surgery center
Cordeiro et al. (42)	Need for user registration; infrastructure limitations in low-resource countries	Development study of the database	Continuous; platform permanently available	Descriptive metrics (number of images, clinical variables); no formal educational tests yet	No formal training	Web-based mammography platform with 169 clinical/radiologic variables; AI and teaching support
Atarere et al. (37)	Lack of references in ChatGPT; misinterpretation in Bing; screening start age not always up to date	Cross-sectional study	Single Q&A session per model	Classification as reliably appropriate/reliably inappropriate/unreliable per ACG guidelines; interrater agreement (89.2%)	No formal training	Academic evaluation of online chat models on colorectal cancer screening
Wang et al. (41)	Possible diagnostic discrepancies; need for human oversight; integration into clinical workflow	Retrospective comparative study	Single evaluation per case	Concordance with medical reports; consistency analysis; Turing test; qualitative CoT review	No formal training	University hospital thyroid imaging/oncology service
Odabashian et al. (36)	Risk of incorrect responses if used without supervision; need for updating and fine-tuning	Cross-sectional study	Point-in-time performance analysis	Percentage of correct answers by item and domain; analysis by diagnostic/treatment/other subcategories	No formal training	Academic oncology training context (ASCO-SEP)
Hesso et al. (47)	Lack of AI training (73% had never used it); structural, regulatory, and trust barriers	Cross-sectional study	No reported	Online questionnaire; statistical analysis; semistructured interviews with thematic analysis	No formal training	Oncology healthcare professionals in 6 European countries
Tomioka et al. (40)	Dependence on video quality; residual misrecognition; need for integration with video systems	Technical validation study	Intraoperative use and brief educational sessions	IoU, Dice; questionnaires on sensitivity/misrecognition; educational utility scale	Brief system demonstration for surgeons and trainees	Hospital with laparoscopic hepatectomy program
Pan et al. (38)	College-level reading difficulty; low actionability; lack of personalization	Cross-sectional study	Single answer-generation session	DISCERN, PEMAT, misinformation scale, Flesch–Kincaid	No formal training	Academic evaluation of chatbot information on cancer
Catanuto et al. (43)	Need for educational validation; technical complexity for non-informatics users	Methodological development study	No reported	Cosine similarity metrics; example semantic queries	No formal training	Academic environment in breast surgery and medical informatics
Görtz et al. (33)	Need to scale sample; integration into routine care; possible selection bias	Descriptive pilot study	Short test period	Questionnaires on use, usability, benefits, and improvements	No formal training	University hospital urology clinic
Heald et al. (44)	Technological barriers (portal/Internet access); need for human follow-up on genetic results	Observational feasibility study	Single chatbot session per patient	Flow metrics through the chat; CCRAT results; genetic test outcomes	Automated education; additional counseling by GCs/GCAs for results	Hospital colonoscopy clinic (Cleveland Clinic)
Chavez-Yenter et al. (45)	Some patients remain uncertain and require human counseling; limited smartphone access	Descriptive feasibility study	Single chat session of ∼10–14 min	Interaction logs; time metrics; number of additional modules consulted; open-ended questions	No formal training	Primary care setting without active cancer, patients eligible for genetic evaluation
Gillan et al. (46)	Concerns about role displacement (e.g., planners), professional responsibility, and AI dependence	Qualitative study with single-profession focus groups	Single session (58 min) per group	Semistructured guide; thematic analysis of transcripts	No formal training	Radiation medicine service in a cancer center introducing AI

Lack of formal training and user readiness is another significant factor identified in the literature. It is noteworthy that most implementations provided minimal or no formal training to end users on the use or interpretation of AI systems. Most studies simply introduced the AI tool without comprehensive user training; for example, several projects explicitly reported that no “formal training” was provided to clinicians or participants (32–37, 41–43, 46, 47). This lack of training can itself be a barrier, as users may be unfamiliar with AI systems or unsure how to respond to AI-generated results (36). According to surveys of oncology professionals in several European countries, over 70% of respondents had never used AI tools, reflecting a significant knowledge gap and highlighting the lack of AI training in oncology education to date (47). points to a lack of AI knowledge among healthcare professionals as a global problem, with clinicians expressing little confidence in AI due to limited exposure and education. Even when some guidance was offered, it tended to be brief. To illustrate this, one educational intervention provided only a short (one-minute) explanatory video about AI to surgeons in training before they interacted with the AI system (39, 45). In their study, they also supported and argued for the feasibility of integrating an AI-powered patient advice chatbot with only a brief introductory session, effectively expecting users to learn on the job. In these cases, the brevity of the training meant that users approached the AI with little preparation, which could exacerbate misunderstandings or misuse. Consistent reports of “informal training” in many implementations (32–35, 37, 41–43, 46) highlight that implementers often underestimated training needs or faced practical limitations in delivering it. In this regard (36, 46, 47), recommend developing specific AI training or onboarding for both clinicians and patients to improve adoption and safe use in the future.

The accuracy of information and the quality of content emerged as critical concerns, especially in contexts where AI was used to generate or deliver educational content to patients or residents. Multiple evaluations revealed that AI-generated cancer information can be prone to errors, outdated guidance, or misinformation if not carefully validated. Two independent cross-sectional studies evaluated AI-produced cancer patient education materials and reported substantial accuracy issues. In study (34), the responses of an AI chatbot on breast cancer were analyzed using standardized tools. The researchers found potentially outdated content and factual errors, highlighting the need for clinical oversight of the AI knowledge base. They measured the readability and quality of the AI-generated content with instruments such as the Patient Educational Materials Assessment Tool (PEMAT) and readability scores, discovering variable integrity and accuracy in the AI responses (35). A study focused on head and neck oncology information found a reliance on automatically generated content and documented instances of incorrect or inconsistent information provided by AI, as well as errors of omission or commission regarding clinical facts. The need to keep AI content up-to-date was exemplified by an implementation targeting colorectal cancer screening guidelines, where the authors observed that the AI did not reflect the latest screening age recommendations, which could confuse patients if not addressed (37). In this case, the team also quantified AI performance using an inter-rater reliability metric, finding approximately 89% agreement with expert responses, but gaps pointed to areas requiring improvement and refinement of the AI's knowledge. Overall, these studies emphasize that content accuracy is a primary concern: whether AI is explaining treatment options or answering patient questions, oncology experts must verify the results (34, 35, 38). incorporated formal content evaluation methods to systematically identify inaccuracies, diversifying (38) in their study, which evaluated the responses of an AI chatbot to cancer-related questions, using a misinformation scoring scale along with readability formulas (such as the Flesch-Kincaid grade level) and cosine similarity measures to compare the AI's responses with reliable sources, thus measuring both the comprehensibility and the fidelity of the information provided. The recurring theme is that quality control mechanisms are needed when integrating AI into the delivery of educational content, due to the risk of misinformation if the AI's results are taken at face value (34, 35, 38).

Other challenges revolve around user acceptance, roles, and ethical considerations in the implementation of AI in oncology settings (46). In a focus group study at a radiology medicine center, participants (including medical physicists and radiation oncologists) expressed uncertainties about professional responsibilities and how to appropriately balance AI assistance with human oversight; they raised questions about accountability and trust, stating that clearer definitions of AI's role in clinical decision-making are needed to feel comfortable integrating it into their practice. This aligns with findings from interviews and surveys of physicians, who frequently cite issues of trust and transparency as barriers (47); presents an international survey that reported hesitation to trust AI recommendations without understanding the logic, reflecting a broader trust gap when AI is viewed as a “black box.” The same study noted organizational and regulatory factors: participants in the multi-country European study pointed to structural and regulatory obstacles such as varying guidelines, lack of regulatory approval, or unclear policies for AI use, which complicate implementation in different healthcare systems. The implementation context also influences acceptance; in this context (45), introduced an AI-powered chatbot for genetic counseling for cancer patients. Researchers found that some patients were reluctant to fully engage with the chatbot, preferring human interaction for sensitive conversations, indicating that human touchpoints remain crucial in contexts such as communicating genetic risks. In the study, their results showed that the application to a limited number of patients actively used the AI advisor, and some questions posed by the AI remained unanswered, suggesting that the technology needed refinement and perhaps a more favorable introduction for patients to trust it and use it effectively.

The study designs and evaluation strategies employed in these investigations reflect the exploratory nature of AI integration in oncology and the need to evaluate both outcomes and process. A variety of study designs were represented, each chosen to suit the questions posed (32) states in its results that traditional experimental designs were relatively rare given the early state of implementations; only one study was a randomized controlled trial that evaluated the impact of an AI tool on patient education in a urology clinic (33, 45) were descriptive or feasibility studies that piloted an AI system and reported initial findings without a control group, while (34, 36, 47) used cross-sectional designs to survey opinions or analyze AI outcomes at a single point in time (41) is a comparative study that compared the diagnostic consistency of an AI with the assessments of radiologists in thyroid oncology imaging. In this case, tools such as a Turing test analogue and case-by-case consistency analysis were used, and they found that, while the AI performed well in many cases, human supervision was still deemed necessary for safety. The duration of the interventions varied widely: some integrations were essentially one-off or short-term exposures, such as (46) a single focus group session lasting approximately one hour in the qualitative study, or a single 10- to 14-minute chatbot interaction in the genetic counseling feasibility study (45), while others involved the continuous use of an AI tool during routine care over a period of time (e.g., using an AI assistant during the diagnostic assessment phase in the clinical trial (32), or a multi-week pilot that integrated AI into the clinical workflow (33). This variation in duration reflects how AI can be introduced as a brief supplement to existing practice or as a sustained component of the care process, each with different challenges. Accordingly, the assessment tools were diverse, aiming to capture both objective outcomes and user perceptions. Quantitative questionnaires were common; for example, a 12-item questionnaire was used to assess knowledge acquisition or understanding following an educational session. AI-assisted training for physicians in training (39) was used, and brief (often customized) surveys were administered to gather user feedback on AI tools in clinical use (32, 38).

In content-focused studies, as mentioned, standardized assessment instruments (such as PEMAT for material quality or readability and misinformation metrics) were applied to objectively rate AI-generated content (34, 35, 38). In contrast, qualitative transcript analysis was employed in studies analyzing communication processes; one study systematically analyzed focus group transcripts to identify themes related to the impact of AI on communication and team workflow in radiation oncology (46) (47) used interview transcripts of healthcare providers across countries to conduct a thematic analysis, uncovering common barriers such as a lack of resources and training in different healthcare settings. Some studies also tracked usage analysis as part of their evaluation, in an implementation of Using an AI symptom checker and risk assessment tool integrated into a clinic's patient portal, researchers collected interaction logs and time data to assess feasibility and patient engagement, and found that while the tool was technically feasible, further follow-up was needed to clarify outcomes for patients (44).

The contextual settings for these AI interventions ranged from patient-oriented applications to clinician-centered tools, and from single-institution local studies to international collaborations, influencing both the challenges encountered and the generalizability of the findings. In patient education and counseling contexts, such as the prostate cancer chatbot trial (32) and the genetic counseling chatbot study (45), the environment required AI to communicate in simple terms and be integrated into the patient's care experience, raising issues of comprehensibility, engagement, and trust from the patient's perspective. Conversely, clinician-oriented AI tools in diagnosis or treatment planning, AI assistance in radiology (41, 42), or surgery (39, 40) had to fit within clinical decision-making workflows and professional standards, highlighting the need for accuracy, efficiency, and a clear delineation of AI responsibilities vs. human responsibilities. Academic and training settings also provided a unique context (39). One study implemented AI in an educational setting for trainee physicians, using it as a teaching aid and then assessing the knowledge gains and attitudes of the trainees. In that academic context, the researchers observed that while AI could convey information quickly, the students' ability to critically evaluate the AI's outputs depended on their pre-existing knowledge and the brief guidance provided, again highlighting the importance of AI literacy in the curriculum (47). Presents broad contextual analyses, such as the multi-country survey of pharmacists and oncologists on AI in practice, which shed light on systemic and cultural factors, noting that resource-rich and resource-limited settings may face different specific barriers, but both share core needs for training and regulatory clarity (42). Explicitly addressed low-resource settings by examining the limitations of an AI platform for mammography in contexts with limited data and infrastructure. It was emphasized that, without adapting AI models to local needs and ensuring that datasets represent diverse populations, the benefits of AI may not reach regions with limited resources. The results provide a global perspective, illustrating that, while the promise of AI in oncology is universal, its implementation must be sensitive to the local context, including resource availability, user readiness, and the structure of the health system.

## 4 Discussion

### 4.1 AI chatbots and language models in oncology education

The reviewed studies highlight the rapid integration of AI-based conversational agents and extensive linguistic models (LLMs) into oncology training, for both patients and professionals. Notably, B (32) developed “PROSCA,” an AI chatbot for prostate cancer counseling, which was tested in a randomized controlled trial with 112 patients. Men who used the PROSCA chatbot reported significantly fewer unmet information needs and a better understanding of their condition than the control group. A previous German pilot, conducted by (33), similarly interacted with 10 men suspected of having prostate cancer and found that almost 90% of users gained knowledge from the chatbot, and all indicated their willingness to reuse and integrate such tools into healthcare (35) used ChatGPT to generate preoperative counseling materials for patients with head and neck cancer, where 5 experienced surgeons considered the information generated by ChatGPT to be comparable in accuracy and comprehensiveness to that of standard online resources, and in approximately half of the cases preferred the AI-generated text; in another study (34) used ChatGPT with a database of frequently asked questions about breast cancer and asked four surgeons to rate the responses; the ChatGPT responses had an average reliability of ∼4/5 and were consistently considered clear and practical.

Beyond customized systems, other studies analyzed the raw performance of LLMs (36). tested ChatGPT-3.5 on over 1,000 oncology exam questions (ASCO-SEP bank). The model achieved an overall accuracy of 56.1%, with variation across domains. Although this score falls below professional exam standards, it indicates that an LLM already encodes substantial oncology knowledge. Another study (38), systematically consulted public AI chatbots, such as ChatGPT, Bing AI, ChatSonic, and Perplexity, using top Google searches for five common cancers. Of the 100 AI-generated responses analyzed, the average DISCERN score was 5/5 (excellent reliability). No overt misinformation was found, although the content was written at a college reading level and scored low for actionability. Patients using AI chatbots for genetic risk education or cancer screening showed high completion rates: for example, in study (44), most patients who initiated the chatbot for Lynch syndrome completed it, and 129 of 487 users underwent genetic testing. In primary care, a chatbot named “BRIDGE” (45) engaged with 30 out of 103 users invited and influenced those who completed the test to undergo genetic cancer testing.

Despite these encouraging results, the quality of the evidence is still developing. Most interventions were single-center pilots with limited participants. The designs were often uncontrolled or qualitative. Chatbots performed well on specific tasks, but it remains unclear whether their benefits persist long-term or across diverse populations. It is crucial that experts emphasize that AI should complement, not replace, professional training. Survey data indicate that many healthcare professionals will only trust AI if it complements human expertise. In practice, healthcare professionals reported that AI-generated educational content should be clearly labeled as guidance. Evidence suggests that AI chatbots and LLMs can enhance patient and healthcare professional training by providing high-quality information and encouraging learner engagement, but these tools currently function best as complements.

The included literature was dominated by favorable findings, with no neutral or adverse educational outcomes reported. This pattern raises concern for publication and selective outcome reporting bias and supports cautious interpretation. Preregistered protocols and transparent reporting of prespecified outcomes, including null findings, would strengthen confidence in future effectiveness conclusions.

### 4.2 Performance improvement and cognitive learning

Beyond information delivery, AI tools have shown promise in improving cognitive performance and reasoning in oncology contexts. For example (41), developed “ThyroAIGuide,” a GPT-4-based platform that analyzes thyroid ultrasound reports with a thought-chain reasoning engine. In retrospective tests of 109 cases, ThyroAIGuide improved report clarity and physician interpretability, aiming to help non-specialists understand the findings, although its diagnostic accuracy remained limited.

In surgical education, AI-assisted tools were linked to performance improvements (40). created an intraoperative guidance system for laparoscopic liver resection that highlighted key hepatic vessels on the surgeon's screen using deep learning segmentation. Although the segmentation accuracy was moderate, ten surgeons in training unanimously rated the AI-enhanced videos as “very useful” for learning anatomy and procedural flow. Similarly, in robotic rectal surgery (39), a U-Net model was trained in Japan to identify autonomic nerves during dissection. Students exposed to this system showed measurable improvements in anatomical recognition, indicating a positive impact on cognitive ability, although long-term outcomes were not monitored.

The role of AI in knowledge acquisition was also assessed using questionnaire-type formats. Studies (35) and (34) showed that AI-generated material can match expert content and may support learning. When evaluated using standardized oncology questions, ChatGPT showed moderate performance, reflecting broad baseline knowledge despite falling below certification-level expectations (36). Some studies also suggested that the accessibility of AI-generated responses could support students' study workflows.

Most studies, including the PROSCA trial (32) and the ChatGPT evaluations (34–36), assessed knowledge or perceptions shortly after the intervention and rarely included control groups or longitudinal follow-up. As a result, it remains unclear whether AI-assisted learning produces durable improvements in knowledge retention, behavioral change, or downstream clinical performance. For example (39), reported improved nerve recognition during training but did not assess whether this translated into subsequent surgical performance. Likewise, studies reporting high expert ratings of ChatGPT-generated content did not consistently link these ratings to objective learning outcomes such as exam performance or patient comprehension (34, 35). Small sample sizes and limited comparator designs further constrain inference, as several findings relied on few evaluators or single-center cohorts (32–36). Current evidence suggests that AI can support cognitive learning, improving comprehension, information retrieval, and selected reasoning tasks, but these gains are primarily short-term and context-specific. Rigorous trials and longitudinal evaluations are needed to establish sustained educational impact. Overall, evidence supports feasibility and acceptability, whereas durable educational effectiveness remains preliminary given limited controls and follow-up.

From an educational perspective, most outcomes mapped to cognitive learning, while psychomotor/procedural skill development was rarely assessed. Viewed through a competency-based lens, the evidence largely addresses early competency targets rather than longitudinal competence indicators such as retention, behavior change, or entrustment.

A notable gap in the literature is the scarcity of AI applications for hands-on training in procedures or technical skills. Apart from a few surgical examples, virtually no studies addressed psychomotor learning. Of the 16 studies reviewed, only (39) explicitly focused on a procedural skill, nerve recognition in robotic rectal surgery. No interventions were found that used AI to teach biopsy techniques, chemotherapy administration, or other technical tasks.

The consequences of this gap are twofold. First, most AI tools in cancer education emphasize information delivery or feedback on tasks such as image interpretation rather than training fine motor skills or high-stakes procedural decision-making required in many cancer interventions. For example (36), discusses ChatGPT's ability to answer questions and support review, but it cannot simulate intraoperative decision-making or complication management. Second, educational studies often lacked robust measures of deep learning. Only a few studies reported quantitative outcomes beyond user satisfaction, and none rigorously assessed skill acquisition against standard training. This limited depth is also reflected in study design, as many reports relied on small pilot cohorts and subjective assessments. Few employed standardized tests or observational checklists capable of detecting subtle improvements. The ChatGPT breast surgery study (34) used surgeon ratings rather than pre- and post-tests, and the surgical skills study (39) did not include a control group or follow-up to assess retention.

### 4.3 Global implementation patterns, barriers, and future directions

Most studies originated in high-resource countries, primarily the United States, with notable contributions from Japan and Germany. Some projects addressed other settings; for example (42), in Brazil, an open-access mammography database was created to support training, explicitly noting the challenges in low-resource settings. This implementation map suggests uneven adoption: North America and parts of Europe/Japan lead the research, while many regions have yet to integrate AI into medical curricula or patient education. Sample sizes and contexts also varied considerably. Some interventions, such as national surveys (47), assessed attitudes across different countries, while others were single-center pilots or departmental programs (32, 44). address patient care chatbots (39, 40), have been tested in clinical portals, while training tools remain confined to the laboratory or operating room.

Technical integration proved difficult: researchers repeatedly reported workflow and infrastructure challenges (32). They noted that their chatbot needed personalization for different patient profiles and better connections with clinical information systems. Similarly, in this context (39), they struggled with their nerve-recognition AI's reliance on high-quality video input, and (40) they encountered segmentation errors that required rigorous validation before clinical use. While efforts are generally made to ensure that AI tools function reliably within established care pathways, these findings underscore that even effective AI models may not reach end users without robust integration into healthcare systems and adequate real-time performance.

Most studies introduced AI tools with minimal guidance, and formal instruction on AI use was often nonexistent: the summary in Table 5 indicates that none of the interventions provided comprehensive training to users. In one surgical study (39), residents received only a one-minute video explanation before using the AI system. Not surprisingly, surveys reveal that many oncology professionals feel ill-prepared: the majority of clinicians surveyed across Europe reported never having used AI tools in practice, and the project described in (47) detected widespread concerns about preparedness and workflow. Adding to this, a study (46) conducted in Canada showed that even those eager to use AI feared “skill erosion” and emphasized that AI should complement, not replace, human expertise. Participants in (46) cautioned that adapting to AI will require new emphases in training, such as interpreting AI results and fostering interdisciplinary collaboration, concluding that human factors are of paramount importance: AI acceptance depends on demystification, building trust, and clear educational support.

Looking ahead, the findings suggest a two-pronged approach for future work. First, researchers should continue the iterative development of effective AI educational tools, rigorously evaluating them. As the authors note, the implementation of AI in oncology training is likely to be gradual and constantly evolving. The technologies themselves are advancing rapidly, as are more effective LLM programs and imaging models, so integration strategies must evolve in parallel. Stakeholders recommend that AI literacy be integrated into health curricula: clinical professionals need formal training on the capabilities and limitations of AI. Second, implementation must take the local context into account. The Brazilian study on mammography (42) illustrated that, without adapting AI models to local data and infrastructure, the benefits may not reach underserved regions. Global equity will require regulatory clarity and resources for implementation in low- and middle-income settings. All the studies (32–46) converge on a common theme: technical excellence alone is not enough; for AI to improve cancer training, it must be accompanied by ongoing AI training, institutional support for training, and a commitment to user-centered design.

### 4.4 Comparative with other studies

The reviews presented in Table 6 address specific applications or general issues. Sacca et al. (48) conducted an exploratory review of machine learning and computer vision models for breast cancer risk prediction and screening. They identified a large number of barriers to adoption and recommended larger datasets, longer follow-up, and AI-based educational tools. Similarly (49), described a US FDA-funded continuing medical education (CME) online course to train pathologists in the assessment of tumor-infiltrating lymphocytes, with the aim of standardizing assessments and improving AI validation.

**Table 6:** Comparison with previous published reviews.

Reference	AI Application	Cancer Type/Clinical Area	Educational/Training Focus	Key Findings
Sacca et al. (48)	Machine/deep learning and computer-vision models for breast cancer risk prediction and screening	Breast cancer—risk prediction and screening	AI in screening	Scoping review identifying some barriers to AI in breast cancer risk prediction. Common barriers include limited external validity (small datasets) and selection bias. Recommended using broader datasets, longer follow-up, and AI-driven education tools to improve risk communication.
Ly et al. (49)	AI/ML algorithms for automating TIL scoring, used to develop diagnostic tools. The study focuses on human training to validate such AI.	Breast cancer—pathology, TIL assessment	Yes—online CME course for pathologists on TIL scoring	Describes a U.S. FDA–supported interactive CME course to train pathologists in scoring stromal TILs, improving the reference standard for AI validation. The course content is designed to increase scoring accuracy and reduce observer variability, ultimately facilitating safe AI adoption.
Caglayan et al. (50)	Large Language Models applied to oncology tasks: clinical decision support, patient education, research data mining (EHR/NGS), and tumor boards	General oncology	LLMs could support education but focus is on clinical/research use	Reviews potential of LLMs in cancer care. Concludes that LLM integration is promising but requires addressing ethical and practical challenges before clinical use.
Lotter et al. (52)	AI applied throughout the cancer clinical “care continuum”, from early detection to treatment and follow-up, with emphasis on integrating AI tools into real clinical practice.	Four prevalent cancers and key clinical domains - imaging detection, assisted diagnosis, therapeutic decisions.	Focuses on the translation clinic, contributing to achieving successful adoption requires adequate training of professionals in the capabilities and limitations of AI.	Structures the advances by cancer type and clinical stage, showing improvements in lesion detection and support for therapeutic decisions, while also highlighting new emerging areas.
Pesapane et al. (53)	Discussion of AI in radiology: emphasizes need for validating AI imaging models across diverse populations to prevent bias.	Breast cancer	Cultural competence and diversity training for radiologists	Reviews global imaging disparities and solutions: advocates scientific mobility and cultural competency training for radiologists to improve access. Emphasizes policy and teleradiology reforms. In the AI era, stresses that imaging AI models must be validated in varied populations to avoid bias and promote equity.
Yang et al. (51)	Latest AI techniques, including multimodal deep learning models and large language models, applied to diagnostic imaging, prognosis, and therapeutic decision-making.	General oncology, with examples in multiple areas	Discusses the introduction of AI in clinical settings and how professionals and patients interact with these tools.	Demonstrate that advanced tools can improve the clarity of reports and medical understanding. AI chatbots have shown high patient engagement rates and the ability to answer oncology questions with moderate reliability.
Farina et al. (56)	Panorama integral de IA, algoritmos de *machine learning* y *deep learning* aplicados a la detección, diagnóstico, tratamiento y predicción de resultados en oncología.	Oncology in general	Mentions the importance of “digital literacy” in oncology in general, but does not describe specific training interventions.	Compiles the current state of AI in oncology, including fundamental principles, current clinical applications, and examples of improvements in clinical practice thanks to AI. It offers an optimistic outlook for the future, highlighting how AI can accelerate cancer research and optimize patient care.
Alabi et al. (58)	AI as a tool to enhance oncologist productivity and reduce workload to combat burnout.	Oncology in general	Training in communication skills, stress management, etc.	Scoping review of 17 studies on preventing oncology burnout. Found both organizational and individual interventions. Notes that AI could mitigate burnout by increasing clinician productivity and efficiency, suggesting AI integration alongside supportive trainings.
Finocchiaro et al. (54)	AI, robotics, and VR/AR in endoscopy simulators for realistic training environments.	Gastrointestinal endoscopy focused in cancer	Simulation-based training for endoscopic procedures	Reviews state of GI endoscopy training simulators, from mechanical models to advanced AI-driven systems. Highlights that recent advances in AI, virtual/augmented reality, and robotics have produced highly realistic simulators.
Talwar et al. (55)	Broad AI applications across oncology: screening, diagnostic imaging, pathology, radiotherapy planning, outcome/treatment prediction, and drug development.	General oncology	None specific (notes lack of AI expertise among clinicians)	Narrative review of AI in oncology: outlines uses in cancer screening, image/pathology diagnosis, radiotherapy planning, and predicting treatment response. Identifies challenges: insufficient validation/generalizability, data limitations, ethical/legal concerns, and a lack of AI training for clinicians. Recommends multidisciplinary collaboration to address these issues.
Shimizu et al. (57)	Diverse applications of AI using deep learning to discover complex patterns.	General oncology	Techniques and tools focused on the understanding and treatment of oncological specialties	Presents numerous recent cases where AI accelerates cancer research and personalized medicine, foreshadowing important advances in the next decade.
Bi et al. (15)	AI-assisted medical image analysis; deep learning algorithms for detection and diagnosis.	Lung, brain, breast cancer and other solid tumors	Focused on clinical advances, it mentions technical training in AI tools in different types of situations related to cancer treatment and prevention.	AI systems matched or surpassed specialists in oncology imaging detection tasks, demonstrating improvements in diagnosis and efficiency. The report provides examples where deep learning solved previously difficult diagnostic problems, boosting accuracy in radiology and oncological pathology.
Our Study	Examines AI in both clinical practice and education across oncology.	General oncology	Focus on integrating AI into medical education	This systematic review synthesizes evidence on how AI is integrated into oncology education and practice worldwide. It identifies effective AI training initiatives, highlights gaps in curricula, and recommends strategies for combining AI educational programs with clinical workflows to improve oncology care.

Other reviews analyzed general AI tools and applications in oncology (50) evaluated LLM models for tasks such as clinical decision support, patient education, research data mining, and tumor board planning, and concluded that LLM integration is promising but requires careful attention to ethical and practical challenges (51). Similarly, they highlighted advanced AI techniques in diagnostic imaging and patient engagement, demonstrating that these tools can improve reporting clarity and patient understanding.

The domains of imaging and procedures feature prominently in the literature (52). They summarized the advances of AI across the cancer care continuum—detection, assisted diagnosis, and treatment planning—highlighting that the integration of AI into practice depends on training clinicians on its capabilities and limitations (53). They focused on AI in radiology, emphasizing the need to validate imaging models in diverse populations to avoid bias; they advocated for culturally sensitive training and teleradiology reforms to promote equitable access (54). They reviewed AI, robotics, and AR/VR in endoscopy simulators, noting that recent advances have led to highly realistic training environments for gastrointestinal procedures (15). They examined deep learning in image analysis for cancers of the lung, brain, and breast, and found that AI systems often match or outperform specialists in detection tasks, underscoring the importance of AI technical training for clinicians.

Several narrative reviews addressed education and workforce issues more generally (55) offered an overview of AI in screening, imaging, pathology, radiotherapy, and prediction, identifying persistent challenges: data limitations, validation gaps, ethical concerns, and a widespread lack of AI expertise among clinicians (56) offered an optimistic perspective on AI, machine learning, and deep learning in oncology, noting the need for “digital literacy” in cancer care, but without detailing specific educational programs (57) showed examples of AI accelerating cancer research and personalized medicine, implying significant advances in the next decade. This study examined burnout prevention in oncology, suggesting that AI could increase productivity and efficiency, but highlighting the continued need for training in communication and stress management.

Compared to these studies, our systematic review uniquely synthesizes the global evidence on AI integration in both oncology education and clinical practice. While previous reviews focus on specific specialties or tools (48–54) or broadly advocate for AI literacy (56, 57), our work spans multiple oncology domains and explicitly links educational initiatives to clinical workflows. We identify effective AI training initiatives and gaps in formal AI curricula worldwide. By combining insights into educational strategies with clinical adoption data, our review expands the more limited scope of previous work and offers a comprehensive, interdisciplinary perspective on AI integration in oncology education and care. For example, we document specific educational programs and curricula implemented in various regions, highlighting a truly global perspective. This broader perspective underscores not only the importance of specialized training, as noted in (49, 53), but also how these initiatives can inform curriculum development and future policies. Our review bridges the gap between the technical promise of AI and the training needs of oncology professionals worldwide, providing a more unified analysis than any single-domain review.

### 4.5 Limitations

In the educational field, few studies have rigorously assessed how formal AI curricula translate into clinical competence, and most educational initiatives lack standardized assessment metrics. These gaps in evidence imply that our synthesis is based primarily on initial experience and expert opinion.

Many AI models rely on large, high-quality datasets; however, available training data are often incomplete or biased. Underreporting, underrepresentation, and heterogeneity in clinical and imaging datasets distort algorithm performance, making it difficult to generalize models across patient populations. AI tools trained on precisely defined populations may perform poorly when deployed in different demographic or technical settings. In practice, healthcare professionals may be reluctant to trust AI-based recommendations without clear explanations, and unexplained model results raise patient autonomy concerns. These technical limitations are widely recognized barriers that likely limit clinical integration. Broader systemic and cultural factors limit AI adoption. Many oncology practices, particularly outside major academic centers, lack the necessary infrastructure, and the upfront costs of data storage and AI software can be prohibitive. Regulatory and policy frameworks for clinical AI tools are still evolving; without clear guidelines on validation and clinical use, implementation is uneven across regions.

A lack of training and conceptual guidance means that many oncologists are unaware of the capabilities and limitations of AI. In turn, this limited familiarity slows its adoption and can distort reports on its effectiveness.

## 5 Conclusion

This systematic review reveals that the integration of AI into oncology education and clinical training remains an emerging but rapidly evolving field. While the number of high-quality empirical studies is still limited, the diversity and recognition of innovation in the current literature suggest a growing transformative potential for AI in oncology. From patient education using chatbots to advanced clinical decision support systems and surgical training, AI technologies are increasingly being integrated into diverse educational and clinical settings in global contexts.

Key findings demonstrate that AI-based tools, particularly LLM models such as ChatGPT and specialized platforms like PROSCA or ThyroAIGuide, have shown promising results in improving cognitive performance, patient engagement, and, to a lesser extent, procedural skills. Most interventions produced positive or statistically significant improvements in comprehension, satisfaction, or diagnostic reasoning, despite notable methodological limitations, such as small sample sizes, a lack of control groups, and short-term outcome assessments. A key trend identified in the studies is the widespread application of conversational agents for both professional and patient-directed training. While these tools have been well-received, particularly in areas such as genetic counseling, screening preparation, and preoperative education, challenges remain regarding content accuracy, outdated information, and the need for robust validation processes before large-scale implementation. Equally important is acknowledging the “black box” phenomenon; many users expressed concerns about transparency, interpretability, and trustworthiness when interacting with AI systems in educational or clinical decision-making contexts.

## Data Availability

The original contributions presented in the study are included in the article/Supplementary Material, further inquiries can be directed to the corresponding author.
